# Efficacy and metabolic outcomes of switching from an INSTI-based triple-drug regimen to dolutegravir/lamivudine in treatment-experienced HIV-1-infected patients: a 48-week real-world study

**DOI:** 10.1080/07853890.2026.2662141

**Published:** 2026-05-12

**Authors:** Wei Zhang, Qisui Li, Changgang Deng, Jing Yuan

**Affiliations:** Division of Infectious Diseases, Chongqing Public Health Medical Center, Chongqing, China

**Keywords:** Treatment-experienced, patients with HIV, INSTI-based triple-drug regimen, switch, simplification, DTG/3tc, efficacy, safety

## Abstract

**Background:**

Real-world data on switching from INSTI-based triple therapy to DTG/3TC in the Chinese population are lacking.

**Methods:**

This retrospective study included 214 treatment-experienced patients with HIV from Chongqing Public Health Medical Center (2021–2022) on an INSTI-based triple-drug regimen for ≥6 months who switched to DTG/3TC for >12 months. Patients with pre-existing metabolic diseases were excluded. Participants were categorized: INSTI+TAF-free (*n* = 100, prior TDF/AZT/ABC), INSTI+TAF (*n* = 56, prior BIC/FTC/TAF), and INSTI+TAF+COBI (*n* = 58, prior EVG/c/FTC/TAF). Virological, immunological, and metabolic parameters were assessed at weeks 24 and 48.

**Results:**

The INSTI+TAF+COBI group was significantly older (p=0.000). Virological suppression rates (<50 copies/mL) were high at switch (93%, 85.7%, 91.4%). The INSTI+TAF+COBI group had lower eGFR and higher TG, TC, HDL, and LDL at baseline (*p* < 0.05). After switching, virological suppression remained high (≥90%) at weeks 24 and 48. CD4+ T-cell increases showed no significant intergroup differences. Metabolic changes differed: TC changes were 0.57 mmol/L (IQR: −0.19–0.91) in INSTI+TAF-free, −0.09 mmol/L (−0.47–0.71) in INSTI+TAF, and −0.25 mmol/L (−0.99–0.33) in INSTI+TAF+COBI. LDL changes were 0.23 mmol/L (−0.23–0.67), −0.15 mmol/L (−0.39–0.01), and −0.18 mmol/L (−0.71–0.10), respectively. TC and LDL changes differed significantly among groups (*p* < 0.05).

**Conclusion:**

Switching to DTG/3TC maintained virological suppression and stable immunological outcomes. Patients previously on TAF-containing regimens demonstrated greater metabolic benefits than those on TAF-free regimens, possibly related to baseline TDF effects, requiring further confirmation.

## Introduction

Long-term antiretroviral therapy is often accompanied by adverse drug reactions (ADRs) and non-AIDS-­defining events (NADEs). These events include cardiovascular, hepatic, and renal diseases; non-AIDS-related malignancies; diabetes; neurocognitive impairments; and bone-related abnormalities [1–3]. These complications inevitably affect patient adherence and quality of life, leading some patients to switch their antiretroviral therapy regimens [4].

In China, the three most commonly used INSTI-based regimens include DTG combined with TDF, AZT, or ABC plus 3TC (INSTI + TAF-free group); BIC/FTC/TAF (INSTI + TAF group); and EVG/c/FTC/TAF (INSTI + TAF + COBI group). However, the long-term use of multiple antiretroviral therapy drugs has raised several concerns, such as pill burden, ADRs, and drug resistance [5,6]. Additionally, some patients who achieve virological suppression may request that their ART regimens be switched due to drug–drug interactions (DDIs), economic burdens, or personal preferences [7,8]. However, real-world data on switching virologically suppressed patients from these regimens to the dual regimen DTG/3TC are lacking in the Chinese population.

Switching treatment-experienced patients to dual simplified regimens is a current focus of research [9]. In China, DTG/3TC is the most commonly used dual simplified regimen, combining the high resistance barrier of DTG with the synergistic effects of 3TC. Multiple studies have demonstrated that the efficacy of DTG/3TC is noninferior to that of triple-drug regimens, such as TDF combined with 3TC/DTG (or Biktarvy) [10,11]. Mechanistically, as long as viral suppression is rapid and durable, the likelihood of the development of resistance is low [12]. Additionally, compared with traditional triple-drug regimens, DTG/3TC reduces drug exposure, thereby decreasing drug-related toxicity and improving patient adherence and quality of life [13]. These three regimens differ in their components (TDF/AZT/ABC vs. TAF vs. TAF+COBI), which may differentially impact metabolic and renal profiles. Understanding post-switch outcomes by prior regimen could guide individualized switching strategies [14].

Therefore, we aimed to evaluate the virological suppression rate, immunological recovery, and metabolic changes (lipids, renal function) at 24 and 48 weeks after switching from different INSTI-based triple regimens to DTG/3TC in treatment-experienced Chinese PLHIV, stratifying by prior regimen type.

### Ethical approval

This study was approved by the Ethics Committee of Chongqing Public Health Medical Center (Approval Number: 2024-082-01-KY). As this was a retrospective study, the requirement for informed consent was waived. The study was conducted in accordance with the Declaration of Helsinki.

### Study population and design

From 2021 to 2022, data were collected from patients with HIV at Chongqing Public Health Medical Center who had been treated with INSTI-based regimens for at least 6 months and subsequently switched to the DTG/3TC regimen for more than 12 months. Patients with preexisting metabolic disorders at baseline were excluded. Virological, immunological, renal, lipid, and glycaemic metabolic differences were observed at 24 and 48 weeks following the switch to the DTG/3TC regimen.

The inclusion criteria were based on the ‘Chinese Guidelines for Diagnosis and Treatment of HIV/AIDS (2021)’ and included the following [15]:

1. HIV-1-infected adults (≥18 years).
2. Virological suppression was defined as HIV RNA <50 copies/mL.

The safety evaluation included assessments of renal function, lipid profiles, glucose levels, and other metabolic parameters.

Exclusion criteria included the following:

1. Coinfection with hepatitis B virus (HBV);
2. Impaired renal function (creatinine clearance rate <50 mL/min).

### Data collection

The baseline demographic and clinical characteristics were extracted from electronic medical records and laboratory databases. These included demographic and clinical data at the time of treatment, such as age, sex, marital status, mode of transmission, duration and number of prior ART regimens, drug resistance testing results, and reasons for switching. Data collected at the start of the treatment changes, as well as at 24 and 48 weeks postswitching, included ART regimens, HIV-RNA viral load (VL), CD4+ T-cell count, SCr levels, Uric Acid(UA) levels, lipid profile, and glucose levels. Adverse events were recorded at each follow-up visit.

### Outcomes of interest

Treatment-experienced patients who met the inclusion and exclusion criteria were categorized into three groups based on their preexisting regimens: the INSTI + TAF-free group, the INSTI + TAF group, and the INSTI + TAF + COBI group. The primary study endpoints included virological suppression rates and changes in CD4+ T-cell counts at weeks 24 and 48 postswitching. The secondary endpoints included assessments of renal function, lipid profiles, and glucose metabolism at weeks 24 and 48 to evaluate metabolic safety.eGFR was calculated using the CKD-EPI equation. renal impairment defined as an eGFR < 90 mL/min/1.73 m^2^, and LLV is defined as a confirmed HIV-1 RNA between 50 and 200 copies/mL.

### Statistical analysis

Statistical analyses were conducted using SPSS version 23.0. Categorical variables are summarized as frequencies and percentages, with group comparisons performed using the χ^2^ test or Fisher’s exact test. Normally distributed continuous variables are reported as the means ± standard deviations (x̅ ± s) continuous variables were analyzed using Kruskal-Wallis test (non-parametric) or one-way ANOVA (parametric) as appropriate. For the primary endpoint of virological suppression, 95% confidence intervals (CIs) were calculated using the Clopper-Pearson exact method. Continuous variables are presented with interquartile ranges (IQR).and were analysed with nonparametric tests. A p value < 0.05 was considered statistically significant.Missing data were handled by last observation carried forward (LOCF) for continuous variables, and actual denominators are reported at each time point. Analyses were per-protocol, including all patients who completed the follow-up visit with available data.

## Results

### Baseline characteristics at the time of switch

**Figure 1.**
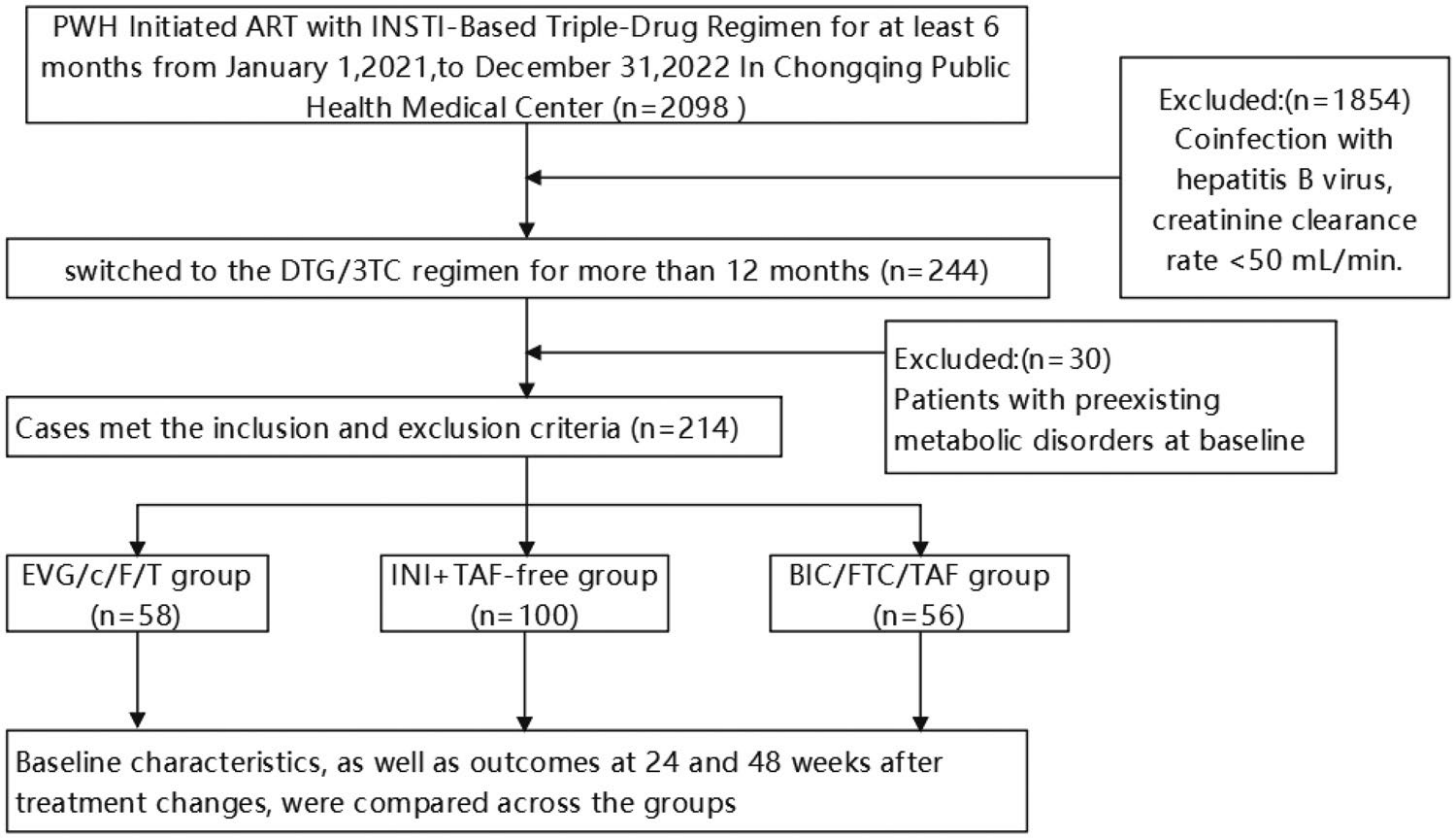
Study flowchart.

Of the 214 patients at baseline, 140 (65.4%) had available data at week 24, and 95 (44.4%) at week 48. Loss to follow-up was due to transfer to other facilities, withdrawal, or not yet reaching the 48-week time point.A total of 214 treatment-experienced patients with HIV who switched from INI-based 3DR regimens to the DTG/3TC regimen were included in the study. The INI+TAF-free group consisted of 100 patients previously treated with the DTG + 3TC + TDF, AZT or ABC regimens. The INI+TAF group included 56 patients who had been on the BIC/FTC/TAF, whereas the INI+TAF+COBI group included 58 patients who had received the EVG/c/FTC/TAF (Figure 1).

At the time of the switch, patients in the INI+TAF+COBI group had a greater mean age (58.4 ± 14.4 years) than did those in the other two groups (INI+TAF-free group: 48.3 ± 15.7 years; INI+TAF group: 46.1 ± 13.3 years; *p* = 0.000). Among the 214 patients, 74 (34.6%) underwent pretreatment drug resistance testing. Each group had one case of NRTI resistance, whereas resistance to INIs was not detected.Reasons for switching varied by group:,simplification or economic reasons were predominant in the INSTI+TAF-free group, while renal impairment, lipid abnormalities, and drug-drug interactions were more common in the TAF-containing groups. Detailed reasons are provided in Table 1.

**Table 1.** Baseline characteristics at the time of switch.

	DTG+TAF-free Group (*n* = 100)	BIC+TAF Group (*n* = 56)	EVG+TAF+COBI Group (*n* = 58)	F/χ^2^ /Z	*P-*value
Male (n, %)	77 (77.0%)	39 (69.6%)	36 (62.1%)	4.048	0.133
Age (years, x̅ ± s)	48.3 ± 15.7	46.1 ± 13.3	58.4 ± 14.4	21.264	0.000
Marital Status					
Single	28 (28.0%)	20 (35.7%)	19 (32.8%)	2.110	0.721
Married	57 (57.0%)	27 (48.2%)	33 (56.9%)		
Divorced or widowed	15 (15.0%)	9 (16.1%)	6 (10.3%)		
Mode of Transmission					
Male-to-Male Sexual Transmission	26 (26.0%)	13 (23.2%)	10 (17.2%)	3.999	0.356
Heterosexual Transmission	71 (71.0%)	43 (76.8%)	48 (82.8%)		
Blood Transfusion/Blood Products	3 (3.0%)	0	0		
Duration of Prior ART (months)	24.0 (14.3–33.0)	22.0 (11.3–67.0)	35.0 (21.8–51.0)	12.758	0.002
Number of Prior ART Regimens	1 (1-1)	2 (1–3)	2 (1–2)	50.791	0.000
Drug Resistance Testing Status					
Test Performed	45	13	16		
Drug Resistance Detected	14 (including 1 case of 3TC resistance)	5 (including 1 case of nucleoside resistance)	4 (including 1 case of nucleoside resistance)		

### Baseline characteristics at the time of treatment Change for each group

At the time of the treatment change, the virological suppression rates for the INI+TAF-free, INI+TAF, and INI+TAF+COBI groups were 93.0%, 85.7%, and 91.4%, respectively. In the INI+TAF-free group, 7 patients (7.0%) exhibited LLV (defined as HIV-1 RNA 50–199 copies/mL). In the INI+TAF group, 8 patients (14.3%) did not achieve virological suppression, including 5 with LLV, 2 with HIV-1 RNA levels between 200–500 copies/mL, and 1 exceeding 1000 copies/mL. In the INI+TAF+COBI group, 5 patients (8.6%) did not achieve virological suppression, including 4 with LLV and 1 with HIV-1 RNA levels between 500–1000 copies/mL. At baseline, no significant differences were observed in UA, SCr, or blood glucose levels among the three groups. However, the eGFR in the INI+TAF+COBI group was significantly lower than that in the other two groups (*p* = 0.000). Additionally, TG, TC, HDL, and LDL levels were significantly greater in the INI+TAF+COBI group than in the other two groups (*p* < 0.05). See Table 2 for details.

**Table 2.** Baseline characteristics at the time of treatment Change for each group.

	DTG+TAF-freeGroup (*n* = 100)	BIC+TAF Group (*n* = 56)	EVG+TAF+COBIGroup (*n* = 58)	X2/Z	*P-*value
CD4 (cells/µL)	314.0 (199.3–443.0)	356.5 (242.3–441.5)	368.0 (230.0–493.0)	2.785	0.248
HIVRNA <50copies/ml (n, %)	93 (93.0%)	48 (85.7%)	53 (91.4%)	2.255	0.318
SCr (umol/L)	80.4 (69.8–95.2)	81.5 (64.9–97.5)	104.5 (73.5–152.7)	2.724	0.256
UA (umol/L)	358.0 (283.0–414.0)	365.5 (311.0–460.0)	376.0 (320.0–466.0)	3.402	0.182
eGFR	96.0 (78.0–110.0)	104.5 (85.5–112.3)	78.0 (56.0–102.3)	17.926	0.000
TG (mmol/L)	1.7 (1.1–2.2)	1.7 (1.2–2.6)	2.2 (1.4–3.4)	9.414	0.009
TC (mmol/L)	4.1 (3.5–4.8)	4.4 (3.6–5.5)	5.3 (4.5–6.3)	27.457	0.000
HDL (mmol/L)	1.1 (0.9–1.3)	1.1 (0.9–1.3)	1.2 (1.0–1.6)	11.028	0.004
LDL (mmol/L)	2.5 (2.0–2.9)	2.7 (2.1–3.2)	2.9 (2.3–3.6)	13.217	0.001
Blood Glucose (mmol/L)	5.7 (5.3–6.3)	5.6 (5.3–6.3)	5.9 (5.3–6.5)	0.943	0.624

### Week 24 changes in parameters

Of the 214 patients at baseline, 140 (65.4%) had available data at week 24, and 95 (44.4%) at week 48. Loss to follow-up was due to transfer to other facilities, withdrawal, or not yet reaching the 48-week time point.After switching to DTG/3TC, lipid trajectories diverged significantly among groups.At week 24 following treatment changes, the virological suppression rates were 90.3% (65/72,95%CI 80.1–96.0%) in the INI+TAF-free group, 96.8% (30/31,95% CI 82.4–99.8%) in the INI+TAF group, and 100% (37/37,95% CI 88.0–100%) in the INI+TAF+COBI group. A total of 8 cases of LLV were observed, including 7 cases in the INI+TAF-free group and 1 case in the BIC/FTC/TAF group. The changes in CD4 counts were 40.0 (−1.0–112.5), 30.5 (−61.3–97.5), and 23.0 (−14.0–110.0) cells/μL in the INI+TAF-free, INI+TAF, and INI+TAF+COBI groups, respectively. No statistically significant differences were noted between the groups (*p* > 0.05). Changes in the SCr, UA, eGFR, blood glucose, and HDL levels were also not significantly different across the groups (*p* > 0.05). After switching to DTG/3TC, TG, TC, and LDL levels decreased from baseline in the INI+TAF+COBI group but increased in the INI+TAF-free and INI+TAF groups. These changes were significantly different among the three groups (*p* < 0.05). See Table 3 for details.

**Table 3.** Changes in parameters from baseline to week 24 post-treatment change.

Changes in Parameters	DTG+TAF-free Group (*n* = 100)	BIC+TAF Group (*n* = 56)	EVG+TAF+COBI Group (*n* = 58)	X2/Z	*P-*value
CD4(cells/µL)	40.0 (−1.0, 112.5)	30.5 (−61.3, 97.5)	23.0 (−14.0-110.0)	0.858	0.651
SCr (umol/L)	−0.4 (−5.7, 6.7)	0.6 (−4.7, 6.4)	1.9 (−4.6-6.7)	0.771	0.680
UA (umol/L)	8 (−24.5-76.5)	−22 (−46, 73)	−7 (−70.5-36.5)	3.569	0.168
eGFR	0 (−6-6)	−0.5 (−4-3.3)	−2.0 (−5-3)	1.428	0.490
TG (mmol/L)	0.37 (−1.7, 0.93)	0.23 (−3.7-1.03)	−0.33 (−1.49, 0.15)	19.602	0.000
TC (mmol/L)	0.59 (0.06–0.97)	0.47 (−0.06-0.80)	−0.21 (−0.98-0.46)	17.459	0.000
HDL (mmol/L)	0.03 (−0.08-0.16)	0.08 (−0.005-0.28)	0.02 (−0.13-0.12)	4.516	0.105
LDL (mmol/L)	0.29 (−0.18-0.52)	0.2 (−0.24-0.38)	−0.05 (−0.39-0.40)	7.285	0.026
Blood Glucose (mmol/L)	0.06 (−0.46-0.71)	0.22 (−0.32-0.79)	0.03 (−0.84-0.57)	1.370	0.504

### Week 48 changes in parameters

At week 48 following treatment changes, the virological suppression rates were 91.3% (42/46, 95% CI 79.7–96.6%) in the INI+TAF-free group, 100% (24/24,95% CI 86.2–100%) in the INI+TAF group, and 96.0% (24/25,95% CI 80.5–99.3%) in the INI+TAF+COBI group. A total of 5 cases of LLV were observed, including 4 cases in the INI+TAF-free group and 1 case in the INI+TAF+COBI group. Additionally, all 3 patients with preexisting 3TC resistance at baseline achieved virological suppression by week 48. The CD4+ T-cell count increased in all groups, but the magnitude of change was not statistically significant (*p* > 0.05). No significant differences in changes in the SCr, UA, eGFR, blood glucose, HDL, or TG levels were detected among the groups (*p* > 0.05). However, patients in the TAF-containing groups presented significant reductions in TC and LDL levels compared with those at baseline. The differences in these changes among the three groups were statistically significant (*p* < 0.05). See Table 4 for details.

**Table 4.** Changes in parameters from baseline to week 48 post-treatment change.

Changes in Parameters	DTG+TAF-free Group (*n* = 100)	BIC+TAF Group (*n* = 56)	EVG+TAF+COBI Group (*n* = 58)	X2/Z	*P-*value
CD4(cells/µL)	34.0 (−9.0, 87.0)	117 (37–191)	73.0 (11.0-149)	3.793	0.150
SCr (umol/L)	−1.75 (−7.5, 4.6)	−2.1 (−17.8, 18.8)	0.35 (−5.3, 8.6)	0.702	0.704
UA (umol/L)	5.5 (−29.3-52.5)	4.5 (−130, 52.3)	−16.5 (−108.5-32.0)	3.053	0.217
eGFR	0 (−6-6.3)	0 (−14.5-4.3)	−1 (−7,3)	1.131	0.568
TG (mmol/L)	0.22 (−0.35, 0.79)	−0.11 (−0.84-3.3)	−0.47 (−0.78, 0.33)	5.265	0.072
TC (mmol/L)	0.57 (−0.19-0.91)	−0.09 (−0.47-0.71)	−0.25 (−0.99-0.33)	6.688	0.035
HDL (mmol/L)	0.04 (−0.10-0.15)	−0.18 (−0.47-0.21)	−0.05 (−0.19-0.13)	2.205	0.332
LDL (mmol/L)	0.23 (−0.23-0.67)	−0.15 (−0.39-0.01)	−0.18 (−0.71-0.10)	9.679	0.008
Blood Glucose (mmol/L)	0.05 (−0.98-1.09)	−0.18 (−1.37-0.35)	−0.14 (−0.52-0.60)	0.474	0.789

## Discussion

For virologically suppressed PLHIV, switching to a dual regimen such as DTG/3TC can reduce pill burden and potential toxicity while maintaining efficacy [16]. In China, integrase inhibitor-based triple-drug regimens are widely used. However, data on the efficacy and safety of transitioning virologically suppressed patients to DTG/3TC are lacking. To the best of our knowledge, this is the first real-world study in China investigating the efficacy and safety of switching treatment-experienced patients with HIV from INI-based triple-drug regimens to DTG/3TC.

Over the past 40 years of HIV treatment, we have now entered the era of INSTI. INSTI-containing regimens, characterized by their high efficacy in viral suppression, have become the cornerstone of international HIV treatment guidelines [17]. In this study, 214 patients on INSTI-based three-drug regimens (INI-based 3DR) were categorized into three groups on the basis of their combination therapies: the INI+TAF-free group, the INI+TAF group, and the INI+TAF+COBI group. Their virological suppression rates (defined as HIV-1 RNA <50 copies/mL) were 93.0%, 85.7%, and 91.4%, respectively, which is consistent with previous conclusions. Regarding the reasons for switching regimens, economic factors and pill burden were the primary considerations in the INI+TAF-free group, whereas adverse drug reactions, including lipid abnormalities, bone metabolism disorders, liver or kidney dysfunction, and DDIs with tuberculosis medications, were the main reasons in the INI+TAF and INI+TAF+COBI groups. These findings suggest that the DTG/3TC dual-drug regimen offers advantages such as reduced pill burden and cost, fewer adverse effects, and minimal drug interactions. LLV was observed in all three groups. Previous studies [18,19] have suggested that LLV may be associated with viral reservoirs, individual variability, drug interactions, or poor treatment adherence. LLV poses risks of treatment failure, transmission, and sustained immune activation and inflammation, which can damage organs and the vasculature, impair immune reconstitution, and negatively impact overall health [20,21]. Thus, regular monitoring and optimization of ART are essential. After switching to the DTG/3TC regimen, the virological suppression rates showed an increasing trend at weeks 24 and 48. CD4+ T-cell counts improved across all groups, and the proportion of patients with LLV decreased. Notably, all three patients with baseline 3TC resistance achieved virological suppression at week 48. These findings highlight the advantages of the DTG/3TC dual-drug regimen in maintaining virological suppression and supporting immune reconstitution after treatment-experienced switches, which is consistent with findings from other studies [22,23].

As the life expectancy of HIV/AIDS patients continues to increase, the risk of NADEs, such as chronic renal insufficiency, has also increased [24]. Additionally, long-term use of antiretroviral drugs is associated with metabolic abnormalities, leading to conditions such as dyslipidaemia, diabetes, and metabolic syndrome, which have garnered increasing attention. In this study, at the time of switching from the INSTI-based 3DR regimen to the DTG/3TC regimen, TG, TC, HDL, and LDL levels were significantly higher in the INI+TAF+COBI group than in the other two groups. By week after treatment changes, TG, TC, and LDL levels in the INI+TAF+COBI group had decreased from baseline, whereas levels in the INI+TAF-free and INI+TAF groups had increased from baseline. At week 48, cholesterol and LDL levels in the INI+TAF+COBI group further decreased, suggesting a clear metabolic benefit of switching to DTG/3TC, particularly in the INI+TAF and INI+TAF+COBI groups compared with the INI+TAF-free group. The divergent lipid changes may be partly explained by the baseline regimens: TDF has a lipid-lowering effect, which could explain the post-switch increases in the INSTI+TAF-free group, whereas TAF and cobicistat may contribute to higher baseline lipids that subsequently improve after discontinuation [25]. However, given the observational nature, these findings should be interpreted cautiously.

In conclusion, this study represents the first real-world investigation of this topic in the Chinese population, featuring long-term follow-up of 48 weeks and comprehensive assessment of virological, immunological, and metabolic parameters.The virological suppression rates and immunological outcomes were well maintained after switching from INSTI-based triple-drug regimens to simplified regimens. Compared with the INI+TAF-free group, the INI+TAF and INI+TAF+COBI groups demonstrated significant metabolic benefits after switching to DTG/3TC, which may be related to the baseline use of TDF in the INI+TAF-free group.These observations suggest that switching to DTG/3TC is a safe and effective option for virologically suppressed patients, and the metabolic impact may depend on the composition of the prior regimen. Larger prospective studies are warranted to confirm these findings and to explore long-term cardiovascular outcomes.

Several limitations should be considered. First, this was an observational, non-randomized study; baseline differences (age, lipid levels, eGFR) among groups may have influenced the metabolic outcomes, and we did not perform multivariable adjustment. Second, the sample size decreased over follow-up, potentially introducing attrition bias. Third, the lack of 95% CIs for primary endpoints in the initial submission has been corrected, but the precision of estimates remains limited by the modest sample size. Larger prospective studies are needed to confirm these findings.

## Conclusion

Switching from INSTI-based triple-drug regimens to a simplified DTG/3TC regimen maintained good virological suppression and immunological outcomes. Compared with the INSTI + TAF-free group, patients in the INSTI + TAF and INSTI + TAF + COBI groups demonstrated significant metabolic benefits after switching to DTG/3TC, which may be associated with baseline TDF-related effects in the INSTI + TAF-free group. However, further studies are needed to validate these findings.

## Data Availability

The data that support the findings of this study can be made available from the corresponding author upon request (yuanjingcq@outlook.Com).
